# 3-(7-Meth­oxy-β-carbolin-1-yl)propionic acid monohydrate

**DOI:** 10.1107/S160053681101350X

**Published:** 2011-04-16

**Authors:** Dong-Mei Dai, Jia-Liang Zhong, Hui-Mei An, Jian-Wei Zou

**Affiliations:** a Key Laboratory for Molecular Design and Nutrition Engineering, Ningbo Institute of Technology, Zhejiang University, 1st Qianhu Nan Road, Ningbo 315100, People’s Republic of China; b Shanghai Institute of Pharmaceutical Industry, 1320 Beijing Road (West), Shanghai 200040, People’s Republic of China; c Beijing Huilongguan Hospital, No.1 Nandian Huilongguan, Changping District, Beijing 100096, People’s Republic of China

## Abstract

In the title compound, C_15_H_14_N_2_O_3_·H_2_O [systematic name: 3-(7-meth­oxy-9*H*-pyrido[3,4-*b*]indol-1-yl)propanoic acid monohydrate], the fused rings make dhedral angles of 0.4 (1), 1.1 (2) and 1.4 (2)°. In the crystal, the water mol­ecule is involved in the formation of three independent hydrogen-bonded chains *via* O—H⋯O and N—H⋯O hydrogen bonds, while the carb­oxy group forms an inter­molecular O—H⋯N hydrogen bond.

## Related literature

For the isolation of the title compound, see: Kardono *et al.* (1991 ▶). For the preparation, see: Kardono *et al.* (1991 ▶). For its pharmacological activity, see: Kuo *et al.* (2003 ▶). For bond-length data, see: Allen *et al.* (1987 ▶).
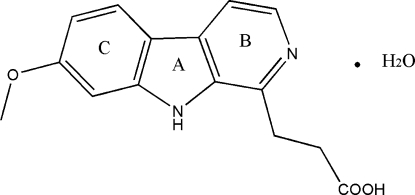


## Experimental

### 

#### Crystal data

C_15_H_14_N_2_O_3_·H_2_O
*M* *_r_* = 288.30
Monoclinic, 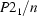

*a* = 4.5114 (1) Å
*b* = 10.8637 (2) Å
*c* = 28.0865 (3) Å
β = 92.414 (1)°
*V* = 1375.31 (4) Å^3^
*Z* = 4
Cu *K*α radiation
μ = 0.85 mm^−1^
*T* = 296 K
0.12 × 0.10 × 0.05 mm

#### Data collection

Bruker APEXII diffractometer
9119 measured reflections
2366 independent reflections
2048 reflections with *I* > 2σ(*I*)
*R*_int_ = 0.022

#### Refinement

*R*[*F* ^2^ > 2σ(*F* ^2^)] = 0.039
*wR*(*F* ^2^) = 0.116
*S* = 1.05
2366 reflections
201 parameters
H atoms treated by a mixture of independent and constrained refinement
Δρ_max_ = 0.49 e Å^−3^
Δρ_min_ = −0.17 e Å^−3^

### 

Data collection: *APEX2* (Bruker, 2005 ▶); cell refinement: *SAINT* (Bruker, 2005 ▶); data reduction: *SAINT*; program(s) used to solve structure: *SHELXS97* (Sheldrick, 2008 ▶); program(s) used to refine structure: *SHELXL97* (Sheldrick, 2008 ▶); molecular graphics: *SHELXTL* (Sheldrick, 2008 ▶); software used to prepare material for publication: *SHELXL97*.

## Supplementary Material

Crystal structure: contains datablocks I, global. DOI: 10.1107/S160053681101350X/mw2003sup1.cif

Structure factors: contains datablocks I. DOI: 10.1107/S160053681101350X/mw2003Isup2.hkl

Additional supplementary materials:  crystallographic information; 3D view; checkCIF report

## Figures and Tables

**Table 1:** Hydrogen-bond geometry (Å, °)

*D*—H⋯*A*	*D*—H	H⋯*A*	*D*⋯*A*	*D*—H⋯*A*
N13—H13*A*⋯O*W*^i^	0.86	1.86	2.7224 (18)	180
O5′—H5′*A*⋯N2^ii^	0.82	1.79	2.5965 (18)	169
O*W*—H*WA*⋯O5′^iii^	0.85 (3)	1.92 (3)	2.7514 (19)	169 (2)
O*W*—H*WB*⋯O4′^iv^	0.87 (3)	1.89 (3)	2.764 (2)	175 (2)

Symmetry codes: (i) 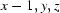; (ii) 
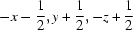
; (iii) 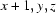; (iv) 
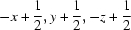
.

**Fig. 1.**
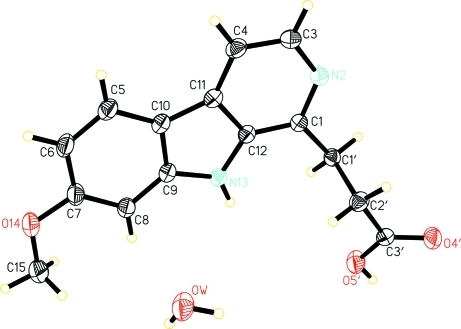
View of the molecule of (I) showing the atom-labelling scheme. Displacement ellipsoids are drawn at the 30% probability level.

**Fig. 2.**
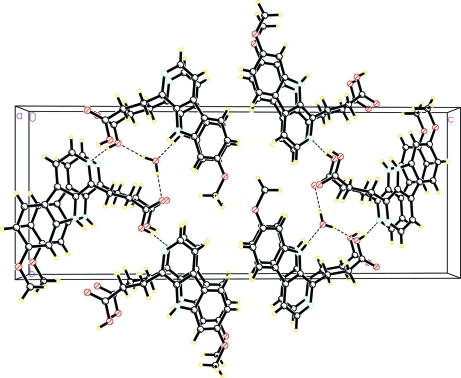
The packing of the title compound,viewed down the a axis. The dashed line indicates the hydrogen bond.

## supplementary crystallographic information

### Comment

The title compound, (I), was isolated from the roots of *Eurycoma longifolia*. It is prepared according to the procedure of Kardono *et al.* (Kardono *et al.*, 1991) and recrystallized from methanol.

Bond lengths and angles are in agreement with reported literature values (Allen *et al.*, 1987). The rings (A, B and C) are each essentially planar with r.m.s deviations of 0.0018 (5) Å, 0.0027 (8)Å and 0.0052 (16) Å, respectively. The dihedral angles between the rings are A/B =1.1 (2)°, A/C = 0.4 (1)° and B/C=1.4 (2)°. The lattice water molecule is involved in formation of three independent hydrogen-bonded chains *via* O—H···O and N—H···O hydrogen bonds while the carboxy group forms an intermolecular O—H···N hydrogen bond (Fig.2 and Table 1). The lattice water molecules could be considered to be a hydrogen-bond bridge which provide further stability to the crystal lattice.

### Experimental

The title compound was prepared according to the procedure of Kardono *et al.*, 1991.Crystals suitable for data collection were obtained by slow evaporation from methanol solution at 283 K over a period of two weeks.

### Refinement

Water H atoms were initially located in a difference Fourier map,and all other H atoms were constrained to an ideal geometry with C—H distances of 0.98 Å and *U*_iso_(H) = 1.2*U*_eq_(C)for CH; 0.97 Å and *U*_iso_(H) = 1.2*U*_eq_ (C)for CH_2_; 0.96 Å and *U*_iso_(H) = 1.5*U*_eq_(C)for CH_3_; and 0.82 Å and *U*_iso_(H) = 1.5*U*_eq_(C)for OH atoms.

### Crystal data

**Table:**

C_15_H_14_N_2_O_3_·H_2_O	*F*(000) = 608
*M**_r_* = 288.30	*D*_x_ = 1.392 Mg m^−^^3^
Monoclinic, *P*2_1_/*n*	Cu *K*α radiation, λ = 1.54178 Å
Hall symbol: -P 2yn	Cell parameters from 3368 reflections
*a* = 4.5114 (1) Å	θ = 3.2–67.6°
*b* = 10.8637 (2) Å	µ = 0.85 mm^−^^1^
*c* = 28.0865 (3) Å	*T* = 296 K
β = 92.414 (1)°	Prism, colourless
*V* = 1375.31 (4) Å^3^	0.12 × 0.10 × 0.05 mm
*Z* = 4	

### Data collection

**Table:**

Bruker APEXII diffractometer	2048 reflections with *I* > 2σ(*I*)
Radiation source: sealed tube	*R*_int_ = 0.022
graphite	θ_max_ = 67.6°, θ_min_ = 3.2°
Detector resolution: 0 pixels mm^-1^	*h* = −4→5
φ and ω scans	*k* = −12→12
9119 measured reflections	*l* = −33→33
2366 independent reflections	

### Refinement

**Table:**

Refinement on *F*^2^	Secondary atom site location: difference Fourier map
Least-squares matrix: full	Hydrogen site location: inferred from neighbouring sites
*R*[*F*^2^ > 2σ(*F*^2^)] = 0.039	H atoms treated by a mixture of independent and constrained refinement
*wR*(*F*^2^) = 0.116	*w* = 1/[σ^2^(*F*_o_^2^) + (0.0672*P*)^2^ + 0.3134*P*] where *P* = (*F*_o_^2^ + 2*F*_c_^2^)/3
*S* = 1.05	(Δ/σ)_max_ = 0.006
2366 reflections	Δρ_max_ = 0.49 e Å^−^^3^
201 parameters	Δρ_min_ = −0.17 e Å^−^^3^
0 restraints	Extinction correction: *SHELXL97* (Sheldrick, 2008), Fc^*^=kFc[1+0.001xFc^2^λ^3^/sin(2θ)]^-1/4^
Primary atom site location: structure-invariant direct methods	Extinction coefficient: 0.0014 (3)

### Special details

**Table:**

Geometry. All e.s.d.'s (except the e.s.d. in the dihedral angle between two l.s. planes) are estimated using the full covariance matrix. The cell e.s.d.'s are taken into account individually in the estimation of e.s.d.'s in distances, angles and torsion angles; correlations between e.s.d.'s in cell parameters are only used when they are defined by crystal symmetry. An approximate (isotropic) treatment of cell e.s.d.'s is used for estimating e.s.d.'s involving l.s. planes.
Refinement. Refinement of *F*^2^ against ALL reflections. The weighted *R*-factor *wR* and goodness of fit *S* are based on *F*^2^, conventional *R*-factors *R* are based on *F*, with *F* set to zero for negative *F*^2^. The threshold expression of *F*^2^ > σ(*F*^2^) is used only for calculating *R*-factors(gt) *etc*. and is not relevant to the choice of reflections for refinement. *R*-factors based on *F*^2^ are statistically about twice as large as those based on *F*, and *R*- factors based on ALL data will be even larger.

### Fractional atomic coordinates and isotropic or equivalent isotropic displacement parameters (Å^2^)

**Table:**

	*x*	*y*	*z*	*U*_iso_*/*U*_eq_	
C1	0.0343 (3)	0.45008 (14)	0.16867 (5)	0.0351 (3)	
N2	0.0495 (3)	0.32718 (11)	0.16361 (5)	0.0402 (3)	
C3	0.2158 (4)	0.27148 (15)	0.13080 (6)	0.0458 (4)	
H3A	0.2177	0.1860	0.1291	0.055*	
C4	0.3807 (4)	0.33780 (15)	0.10014 (6)	0.0438 (4)	
H4A	0.4925	0.2985	0.0776	0.053*	
C5	0.7128 (4)	0.57535 (16)	0.04262 (6)	0.0443 (4)	
H5A	0.7804	0.5050	0.0276	0.053*	
C6	0.8037 (4)	0.68936 (17)	0.02872 (6)	0.0487 (4)	
H6A	0.9324	0.6962	0.0039	0.058*	
C7	0.7055 (4)	0.79694 (16)	0.05145 (6)	0.0460 (4)	
C8	0.5138 (4)	0.79159 (15)	0.08833 (6)	0.0427 (4)	
H8A	0.4502	0.8624	0.1035	0.051*	
C9	0.4200 (3)	0.67511 (14)	0.10178 (5)	0.0374 (4)	
C10	0.5163 (3)	0.56590 (14)	0.07978 (5)	0.0381 (4)	
C11	0.3771 (3)	0.46622 (14)	0.10355 (5)	0.0370 (3)	
C12	0.2008 (3)	0.52071 (14)	0.13862 (5)	0.0350 (3)	
N13	0.2299 (3)	0.64599 (12)	0.13699 (4)	0.0381 (3)	
H13A	0.1437	0.6978	0.1550	0.046*	
O14	0.8192 (3)	0.90335 (12)	0.03401 (5)	0.0602 (4)	
C15	0.7305 (6)	1.01607 (19)	0.05520 (8)	0.0734 (7)	
H15A	0.8250	1.0837	0.0399	0.110*	
H15B	0.7877	1.0156	0.0885	0.110*	
H15C	0.5191	1.0248	0.0514	0.110*	
C1'	−0.1536 (3)	0.50276 (13)	0.20627 (5)	0.0364 (4)	
H1'A	−0.3104	0.4449	0.2129	0.044*	
H1'B	−0.2458	0.5780	0.1944	0.044*	
C2'	0.0251 (4)	0.53087 (15)	0.25274 (6)	0.0437 (4)	
H2'A	0.0966	0.4545	0.2670	0.052*	
H2'B	0.1957	0.5811	0.2458	0.052*	
C3'	−0.1657 (4)	0.59823 (14)	0.28756 (5)	0.0416 (4)	
O4'	−0.2512 (4)	0.54636 (12)	0.32330 (4)	0.0655 (4)	
O5'	−0.2315 (4)	0.70783 (11)	0.27682 (5)	0.0618 (4)	
H5'A	−0.3268	0.7384	0.2981	0.25 (3)*	
OW	0.9592 (4)	0.81052 (13)	0.19395 (6)	0.0690 (5)	
HWA	0.894 (5)	0.789 (2)	0.2205 (10)	0.081 (8)*	
HWB	0.882 (5)	0.883 (3)	0.1889 (9)	0.076 (7)*	

### Atomic displacement parameters (Å^2^)

**Table:**

	*U*^11^	*U*^22^	*U*^33^	*U*^12^	*U*^13^	*U*^23^
C1	0.0370 (8)	0.0361 (8)	0.0322 (7)	−0.0001 (6)	0.0022 (6)	−0.0003 (6)
N2	0.0474 (8)	0.0356 (7)	0.0381 (7)	0.0008 (5)	0.0062 (6)	−0.0012 (5)
C3	0.0572 (10)	0.0358 (8)	0.0450 (9)	0.0031 (7)	0.0082 (7)	−0.0047 (6)
C4	0.0489 (9)	0.0433 (9)	0.0397 (8)	0.0070 (7)	0.0089 (7)	−0.0061 (6)
C5	0.0431 (9)	0.0531 (10)	0.0374 (8)	0.0014 (7)	0.0097 (7)	−0.0048 (7)
C6	0.0482 (10)	0.0627 (11)	0.0363 (8)	−0.0041 (8)	0.0146 (7)	0.0008 (7)
C7	0.0487 (9)	0.0521 (10)	0.0375 (8)	−0.0068 (7)	0.0064 (7)	0.0072 (7)
C8	0.0483 (9)	0.0419 (9)	0.0383 (8)	−0.0002 (7)	0.0084 (7)	0.0012 (6)
C9	0.0384 (8)	0.0422 (8)	0.0318 (7)	0.0006 (6)	0.0049 (6)	0.0006 (6)
C10	0.0376 (8)	0.0443 (9)	0.0326 (7)	0.0012 (6)	0.0039 (6)	−0.0012 (6)
C11	0.0371 (8)	0.0411 (8)	0.0330 (7)	0.0028 (6)	0.0029 (6)	−0.0026 (6)
C12	0.0362 (8)	0.0372 (8)	0.0316 (7)	0.0019 (6)	0.0033 (6)	−0.0009 (6)
N13	0.0440 (7)	0.0350 (7)	0.0362 (6)	0.0021 (5)	0.0120 (5)	−0.0008 (5)
O14	0.0749 (9)	0.0543 (8)	0.0530 (7)	−0.0113 (6)	0.0232 (6)	0.0087 (6)
C15	0.1093 (18)	0.0520 (12)	0.0610 (12)	−0.0196 (11)	0.0274 (12)	0.0019 (9)
C1'	0.0378 (8)	0.0352 (8)	0.0367 (8)	−0.0002 (6)	0.0081 (6)	0.0004 (6)
C2'	0.0472 (9)	0.0451 (9)	0.0389 (8)	0.0062 (7)	0.0038 (7)	−0.0023 (7)
C3'	0.0501 (9)	0.0387 (8)	0.0365 (8)	−0.0028 (6)	0.0061 (7)	−0.0041 (6)
O4'	0.1062 (11)	0.0451 (7)	0.0477 (7)	−0.0034 (7)	0.0302 (7)	−0.0001 (5)
O5'	0.0916 (10)	0.0433 (7)	0.0528 (8)	0.0155 (6)	0.0297 (7)	0.0040 (5)
OW	0.1082 (13)	0.0425 (7)	0.0594 (9)	0.0149 (7)	0.0382 (8)	0.0023 (6)

### Geometric parameters (Å, °)

**Table:**

C1—N2	1.3447 (19)	C10—C11	1.431 (2)
C1—C12	1.385 (2)	C11—C12	1.421 (2)
C1—C1'	1.4955 (19)	C12—N13	1.3683 (19)
N2—C3	1.355 (2)	N13—H13A	0.8600
C3—C4	1.367 (2)	O14—C15	1.426 (2)
C3—H3A	0.9300	C15—H15A	0.9600
C4—C11	1.399 (2)	C15—H15B	0.9600
C4—H4A	0.9300	C15—H15C	0.9600
C5—C6	1.367 (2)	C1'—C2'	1.535 (2)
C5—C10	1.401 (2)	C1'—H1'A	0.9700
C5—H5A	0.9300	C1'—H1'B	0.9700
C6—C7	1.412 (2)	C2'—C3'	1.518 (2)
C6—H6A	0.9300	C2'—H2'A	0.9700
C7—O14	1.364 (2)	C2'—H2'B	0.9700
C7—C8	1.378 (2)	C3'—O4'	1.228 (2)
C8—C9	1.392 (2)	C3'—O5'	1.261 (2)
C8—H8A	0.9300	O5'—H5'A	0.8200
C9—N13	1.3728 (19)	OW—HWA	0.85 (3)
C9—C10	1.414 (2)	OW—HWB	0.87 (3)
			
N2—C1—C12	117.01 (13)	N13—C12—C1	128.81 (13)
N2—C1—C1'	119.18 (13)	N13—C12—C11	109.50 (13)
C12—C1—C1'	123.80 (13)	C1—C12—C11	121.69 (14)
C1—N2—C3	123.20 (13)	C12—N13—C9	108.49 (12)
N2—C3—C4	121.64 (15)	C12—N13—H13A	125.8
N2—C3—H3A	119.2	C9—N13—H13A	125.8
C4—C3—H3A	119.2	C7—O14—C15	117.49 (14)
C3—C4—C11	118.33 (14)	O14—C15—H15A	109.5
C3—C4—H4A	120.8	O14—C15—H15B	109.5
C11—C4—H4A	120.8	H15A—C15—H15B	109.5
C6—C5—C10	119.07 (15)	O14—C15—H15C	109.5
C6—C5—H5A	120.5	H15A—C15—H15C	109.5
C10—C5—H5A	120.5	H15B—C15—H15C	109.5
C5—C6—C7	121.16 (15)	C1—C1'—C2'	112.56 (12)
C5—C6—H6A	119.4	C1—C1'—H1'A	109.1
C7—C6—H6A	119.4	C2'—C1'—H1'A	109.1
O14—C7—C8	124.26 (16)	C1—C1'—H1'B	109.1
O14—C7—C6	114.16 (15)	C2'—C1'—H1'B	109.1
C8—C7—C6	121.58 (15)	H1'A—C1'—H1'B	107.8
C7—C8—C9	116.74 (15)	C3'—C2'—C1'	110.62 (13)
C7—C8—H8A	121.6	C3'—C2'—H2'A	109.5
C9—C8—H8A	121.6	C1'—C2'—H2'A	109.5
N13—C9—C8	127.69 (14)	C3'—C2'—H2'B	109.5
N13—C9—C10	109.52 (13)	C1'—C2'—H2'B	109.5
C8—C9—C10	122.79 (14)	H2'A—C2'—H2'B	108.1
C5—C10—C9	118.65 (14)	O4'—C3'—O5'	123.37 (15)
C5—C10—C11	134.97 (15)	O4'—C3'—C2'	120.80 (15)
C9—C10—C11	106.38 (13)	O5'—C3'—C2'	115.82 (14)
C4—C11—C12	118.13 (14)	C3'—O5'—H5'A	109.5
C4—C11—C10	135.75 (14)	HWA—OW—HWB	104 (2)
C12—C11—C10	106.11 (13)		
			
C12—C1—N2—C3	−0.5 (2)	C5—C10—C11—C12	−179.98 (18)
C1'—C1—N2—C3	−179.45 (15)	C9—C10—C11—C12	0.48 (17)
C1—N2—C3—C4	−0.2 (3)	N2—C1—C12—N13	−178.44 (14)
N2—C3—C4—C11	0.6 (3)	C1'—C1—C12—N13	0.4 (2)
C10—C5—C6—C7	0.6 (3)	N2—C1—C12—C11	0.8 (2)
C5—C6—C7—O14	179.06 (15)	C1'—C1—C12—C11	179.69 (14)
C5—C6—C7—C8	−0.3 (3)	C4—C11—C12—N13	178.96 (13)
O14—C7—C8—C9	−179.74 (15)	C10—C11—C12—N13	−0.36 (17)
C6—C7—C8—C9	−0.5 (3)	C4—C11—C12—C1	−0.4 (2)
C7—C8—C9—N13	−179.63 (16)	C10—C11—C12—C1	−179.75 (14)
C7—C8—C9—C10	0.9 (2)	C1—C12—N13—C9	179.43 (15)
C6—C5—C10—C9	−0.3 (2)	C11—C12—N13—C9	0.09 (17)
C6—C5—C10—C11	−179.79 (17)	C8—C9—N13—C12	−179.34 (16)
N13—C9—C10—C5	179.93 (14)	C10—C9—N13—C12	0.22 (17)
C8—C9—C10—C5	−0.5 (2)	C8—C7—O14—C15	−0.7 (3)
N13—C9—C10—C11	−0.44 (17)	C6—C7—O14—C15	−179.95 (18)
C8—C9—C10—C11	179.14 (15)	N2—C1—C1'—C2'	95.18 (16)
C3—C4—C11—C12	−0.3 (2)	C12—C1—C1'—C2'	−83.68 (18)
C3—C4—C11—C10	178.79 (18)	C1—C1'—C2'—C3'	173.08 (13)
C5—C10—C11—C4	0.9 (3)	C1'—C2'—C3'—O4'	108.25 (18)
C9—C10—C11—C4	−178.66 (18)	C1'—C2'—C3'—O5'	−70.68 (19)

### Hydrogen-bond geometry (Å, °)

**Table:**

*D*—H···*A*	*D*—H	H···*A*	*D*···*A*	*D*—H···*A*
N13—H13A···OW^i^	0.86	1.86	2.7224 (18)	180
O5'—H5'A···N2^ii^	0.82	1.79	2.5965 (18)	169
OW—HWA···O5'^iii^	0.85 (3)	1.92 (3)	2.7514 (19)	169 (2)
OW—HWB···O4'^iv^	0.87 (3)	1.89 (3)	2.764 (2)	175 (2)

Symmetry codes: (i) *x*−1, *y*, *z*; (ii) −*x*−1/2, *y*+1/2, −*z*+1/2; (iii) *x*+1, *y*, *z*; (iv) −*x*+1/2, *y*+1/2, −*z*+1/2.
